# Annual Address

**Published:** 1882-08

**Authors:** G. W. Fuller

**Affiliations:** Des Moines, Iowa


### ANNUAL ADDRESS.

Given before the Iowa State Dental Society by the President, G. W. FULLER, of Des Moines, Iowa.

After a few words of greeting and encouragement, President Fuller spoke as follows:

"I have arranged a few thoughts in the form of an address, not with an idea of originality of matter, but in such a way as to refresh the minds of many, and possibly to instruct and encourage others who have more recently enrolled themselves in the ranks of our profession. If I succeed in this, my object will be accomplished, and I shall be satisfied with the result. It is probable that at an early day some of the human family began to require the services of a dentist, but at what particular date that service was first needed, is not known. It has been asserted by some writers of recent date, that gold fillings and other evidences of ancient dentistry have been seen in the teeth of Egyptian mummies supposed to be of great antiquity, but it has been a question whether the circumstance was of itself an evidence of ancient dentistry or of the modern origin of the individual mummy, since it seems to be a well established fact that a lively and remunerative business has recently sprung up in that city of wonders, the French capital, which consists in the manufacture of an article to imitate those well preserved Egyptians. Esculapius, third son of Arsippus, according to mythology, was the first to teach how to extract teeth, but we are left in the dark as to the agency used in those operations. Probably instead of "nitrous oxide" as an anæsthetic, and modern forceps coupled with muscular force, he, like Jupiter, used thunderbolts for all such difficult undertakings. However that may be, we have running all along down through the annals of history an occasional glimpse of the various methods used in the treatment of the numerous diseases of the oral cavity, and especially the teeth. At times it has been, by some sort of divination, incantation or charm, such as wearing amulets or some equally superstitious means.

We find among the Chinese, those children of the sun, who, according to their literature have forgotten more than all the outside barbarians of other nations have ever learned up to the present time. Tooth doctors, who use medicine to kill the worm as they call it, in some troublesome masticator, which they claim is doing all the mischief. Also among all the Indian tribes of America, medicine men are found who treat the teeth by the use of some herb, root or spell—thus demonstrating to my mind that dentistry, like medicine, has been a child of slow growth through the long ages of the past. Herodotus, who was born about the year 484 B.C., in his writings refers to dentistry in such a manner as to demonstrate that he knew something of it in his day. And thus along down through history we occasionally see some reference to dentistry or dental operations, as practiced in a very crude manner, often by the blacksmith or jeweler, but more generally by the barber, who up to the present day in many parts of Germany, is noted for his skill in extracting teeth. About one hundred years ago, at the close of the American revolution, a man by the name of Joseph Flagg commenced the practice of dentistry in Rhode Island. He held a major's commission in the army, and began practice some time in 1782. I do not know that he was a relative of the present J. Foster Flagg, of Philadelphia, the disciple of "the new departure," but, as far as I have been able to learn, he was the first American dentist. In 1820 there were in this country one hundred dentists, but up to within the last forty years, or a little more, dentistry had assumed no place as a specialty separated from general surgery. Since that time, however, there has been a wonderful, and, I might almost say, a magical change in our progress to the front rank of an acknowledged scientific profession, with all the advantages of special schools and colleges possessing merits of a high order, and that, too, while many of the men still live who have been the most active in bringing about this desirable result. Other grand and noble spirits have gone to "that bourne from whence no traveler returns," but their glorious work still remains as a monument to active and useful lives devoted in a great measure to the good of those who shall come after them.

In 1858, there were four thousand dentistspracticing in this country, and now it is estimated that we have at least thirteen thousand, and that they are using half a ton of pure gold and one hundred thousand dollars worth of cheaper materials annually. There are also made in the United States every year three million artificial or porcelain teeth mounted on the various bases, and still not one-half of the people who need them avail themselves of their services. Statistics also inform us that only about one person in eight can be found with perfect teeth. What a vast multitude of patients is presented to the mind of the intelligent dentist, many millions in this country daily needing the services of skilled dental surgeons to relieve them of present pain and prevent their teeth from destruction in the near future. No wonder that men of genius and ability are being developed in our ranks. when there is such a broad field open to them for active and useful labor; a field, however, that can not be successfully occupied without many trials and conflicts, often meeting with reverses and *occasionally* a triumph. My heart goes out in sympathy toward the energetic young man who is just starting out in our profession with a full determination to succeed, regardless of any probable opposition that may intervene to retard his progress, if within the compass of human endurance and perseverance to do so, when I think of the herculean task before him, and how many days, months and years of close application and assiduous study and labor is allotted to him ere he can reasonably expect to attain that practical success which all really ambitious and meritorious young men look forward to with so much anticipation. There is no doubt of the fact that many an honest and worthy young man who contemplates making dentistry his future occupation does not comprehend or fully appreciate the importance of a thorough education, either scientific or literary, as a foundation upon which to build a professional character and standing in the field of his future practice. All good and true men among us desire to see a continual advancement and elevation of this our chosen profession, and I see no other way to accomplish such a desirable result but by properly educating those young men who in a few years at the farthest must take our places in the ranks of active and aggressive labor, and carry on to a still higher level the work which our predecessors began, and we in our turn are laboring so enthusiastically to advance. Some of the older men of our Society can look back to the time when we had no dental colleges or journals to disseminate dental knowledge throughout the length and breadth of the land, and when what little was known on the subject was guarded as professional secrets, to be imparted only to a favored few, and then for a money consideration That was a dark and gloomy time to the young dentist in comparison with the present, when we have so many facilities for obtaining professional knowledge open to us, among which are the dental colleges, journals, dental societies and conventions, each being a power in its particular sphere of action. We know that occasionally a man of exceptional energy has eminently succeeded without a college or preparatory education; but such men have labored under great difficulties and embarrassments because of the want of early advantages that almost every young man of the present day has within his grasp. Says an eminent writer on dentistry: "It will not long answer that a dentist be merely an expert operator; he must understand the structure and functions of the parts with which he deals, regional and systemic relations, their pathological liabilities, the causes of aberrations and the means and methods of restoring abnormal to healthy conditions." It will be impossible for any one to reach that ideal of dentistry without laborious study and close application for a term of years, at least, and that should be in early life when his mind being fresh and clear is easily impressed. It is then he can build for the future—laying the foundation of that structure which he is to rear in after life deep, and strong, so that it will resist the winds and storms of opposition and adversity, and be like the building we read of in the good book, which "was founded upon a rock." That "knowledge is power" is the old saying but a true one. It is true in every department in life. If a man possesses an intelligent mind, well stored with useful knowledge, he will make an impression upon his fellow men in the community in which he lives, and probably upon the surrounding world, no matter how modest and retiring he may be in his nature and disposition; his influence will be felt and appreciated wherever he is known. We can not all write and speak alike well, but we can each one of us accomplish much good by doing our duty in any position that circumstances may place us. Our Society is composed of various elements. One member may excel as a speaker, another as a writer, and a third as an operator or demonstrator, and so you see that we may each be a mutual benefit to one another. Let us also remember that we are something more than dentists, that we are *men*, and that we each occupy individual positions in society, where by throwing our influence in the proper direction, we can do great good and be honored and respected by all who know us, and live such lives that when we lay down our work it may be said of each of us that the world is the better for his having lived in it.

---

ERRATUM.—In Register for July in letter signed "A Close Observer," read in twelfth line from top of page 354, (M.D.S.) instead of (M.D )

